# From Aggregates to Porous Three-Dimensional Scaffolds through a Mechanochemical Approach to Design Photosensitive Chitosan Derivatives

**DOI:** 10.3390/md17010048

**Published:** 2019-01-10

**Authors:** Kseniia N. Bardakova, Tatiana A. Akopova, Alexander V. Kurkov, Galina P. Goncharuk, Denis V. Butnaru, Vitaliy F. Burdukovskii, Artem A. Antoshin, Ivan A. Farion, Tatiana M. Zharikova, Anatoliy B. Shekhter, Vladimir I. Yusupov, Peter S. Timashev, Yury A. Rochev

**Affiliations:** 1 Institute for Regenerative Medicine, Sechenov University, 8-2 Trubetskaya st., Moscow 119991, Russia; a-kurkov@yandex.ru (A.V.K.); butnaru.dw@gmail.com (D.V.B.); antoshin.art@gmail.com (A.A.A.); zharikova.tm@gmail.com (T.M.Z.); a.shehter@yandex.ru (A.B.S.); timashev.peter@gmail.com (P.S.T.); yury.rochev@nuigalway.ie (Y.A.R.); 2 Institute of Photonic Technologies, Research center “Crystallography and Photonics”, Russian Academy of Sciences, 2 Pionerskaya st., Troitsk, Moscow 108840, Russia; iouss@yandex.ru; 3 Enikolopov Institute of Synthetic Polymer Materials, Russian Academy of Sciences, 70 Profsoyuznaya st., Moscow 117393, Russia; akopova@ispm.ru (T.A.A.); duna2011@yandex.ru (G.P.G.); 4 Baikal Institute of Nature Management, Siberian Branch of the Russian Academy of Sciences, 6 Sakhyanovoy st., Ulan-Ude 670047, Russia; burdvit@mail.ru (V.F.B.); fariv@mail.ru (I.A.F.); 5 Institute for Urology and Reproductive Health, Sechenov University, 2-1 Bolshaya Pirogovskaya st., Moscow 119435, Russia; 6 Semenov Institute of Chemical Physics, Russian Academy of Sciences, 4 Kosygina st., Moscow 119991, Russia; 7 National Centre for Biomedical Engineering Science, National University of Ireland, Galway (NUI Galway), University Road, Galway H91 TK33, Ireland

**Keywords:** mechanochemical synthesis, chitosan, laser stereolithography, long-term stability, scaffold, tissue reaction

## Abstract

The crustacean processing industry produces large quantities of waste by-products (up to 70%). Such wastes could be used as raw materials for producing chitosan, a polysaccharide with a unique set of biochemical properties. However, the preparation methods and the long-term stability of chitosan-based products limit their application in biomedicine. In this study, different scale structures, such as aggregates, photo-crosslinked films, and 3D scaffolds based on mechanochemically-modified chitosan derivatives, were successfully formed. Dynamic light scattering revealed that aggregation of chitosan derivatives becomes more pronounced with an increase in the number of hydrophobic substituents. Although the results of the mechanical testing revealed that the plasticity of photo-crosslinked films was 5–8% higher than that for the initial chitosan films, their tensile strength remained unchanged. Different types of polymer scaffolds, such as flexible and porous ones, were developed by laser stereolithography. In vivo studies of the formed structures showed no dystrophic and necrobiotic changes, which proves their biocompatibility. Moreover, the wavelet analysis was used to show that the areas of chitosan film degradation were periodic. Comparing the results of the wavelet analysis and X-ray diffraction data, we have concluded that degradation occurs within less ordered amorphous regions in the polymer bulk.

## 1. Introduction

Formation of waste by-products from commercial fish and seafood production is a topical problem nowadays. For example, in the case of the crustacean processing industry, the amount of waste by-products reaches 50–70% of the raw material [1]. Such wastes require additional economic costs for their disposal and, therefore, the industry should pay close attention to the waste recycling process. 

Sea crustaceans are known to contain a large amount of chitin, a polysaccharide which is the second most abundant biopolymer after cellulose. It is also found in the exoskeleton of insects, cell walls of fungi, and in green algae [2]. Partial deacetylation of chitin results in the formation of chitosan [3], a biopolymer with a unique set of biological and physicochemical properties. In addition to biodegradability and biocompatibility, chitosan is a non-immunogenic material, which also demonstrates antifungal and antimicrobial activities [4], with an almost absent reaction to a foreign body, no formation of a fibrous capsule [5], and the ability to penetrate through the intestinal barrier [6]. Free amino groups give chitosan many specific properties, distinguishing it from chitin. The amino groups of the d-glucosamine residues can be protonated, and the resulting polycation can subsequently form ionic complexes with various proteins, lipids, DNA and negatively-charged synthetic polymers [2]. Moreover, such polycations have the ability to interact with the negative charges of the cell surface [7]. Materials based on chitosan and its derivatives may be presented in different physical forms. Their mucoadhesive properties allow them to be used as excipients for the preparation of buccal, vaginal and nasal dosage forms [8,9].

Despite extensive renewable sources of chitosan and its universal properties, there are practically no available pharmaceutical products based on this biopolymer. This fact has several explanations. First, chitosan is sensitive to storage and processing conditions. Significant heating or cooling can cause stress to its structure and cause polymer degradation or oxidative transformation of its functional groups [8]. It limits the number of reagents suitable for chemical modification of chitosan. In the numerous studies devoted to the application of chitosan-based materials in the pharmaceutical and biomedical fields, chitosan and its derivatives were obtained mainly through a classical chemical synthesis [10,11,12,13]. Application of toxic solvents as well as a low final product yield do not allow the extrapolation of the synthesis schemes developed in the scientific studies onto an industrial scale.

There is another reason limiting the application of chitosan and its derivatives in the pharmaceutical industry and biomedicine. Being a natural polymer, chitosan has highly variable properties. Its mean molecular weight, molecular weight distribution, degree of deacetylation, and purity are highly dependent on the production methods, as well as on the choice of the raw material source [14]. These characteristics affect the physicochemical (e.g., viscosity, solubility) and biological (e.g., biodegradability, stability) properties of chitosan and its derivatives.

Long-term stability is one of the problems associated with the introduction of drug delivery systems into a wider clinical application. Chitosan-based delivery systems must be thermally, chemically, and mechanically stable thereby maintaining the effectiveness of the dosage form for a long time [15]. Several strategies to increase the long-term stability of chitosan-based products are known: polymer-analogous transformations of functional groups, application of stabilizing agents, and physical and chemical cross-linking [8,16,17].

In our study, we used the technique of processing solid mixtures which combines chemical reagents, pressure and shear stress. This method is known as mechanochemical synthesis [18,19,20]. Compared to the conventional solvent-based chemical synthesis, mechanochemical synthesis is a convenient and effective approach to targeted chemical modification of non-melting or poorly soluble polysaccharides, since it does not require melting the reaction mixtures [21,22]. Mechanochemical synthesis can be adopted to different production conditions as well as scaled and, therefore, this method has been employed across a number of industries including food, polymer, and pharmaceutical manufacturing [23]. Currently, there is a global trend towards using environmentally friendly and safe processes to modify medical polymers. Mechanochemical synthesis has attracted the attention of researchers in the field of biomedical materials science, since it does not require solvents, catalysts, and initiators of chemical processes, thus reducing the negative impact on the environment. As a result, the economic costs of solvent disposal and purification of synthesized medical polymers are excluded.

In our study, we used the same extruder either for alkaline deacetylation of chitin or for its modification with allyl groups on a pilot-industrial scale. Introduction of hydrophobic unsaturated substituents allows us not only to increase the long-term stability of chitosan-based materials, but also to obtain a photosensitive biopolymer. When exposed to ultraviolet and laser radiation, it can crosslink and form stable three-dimensional networks.

Thus, the presented work is aimed at determining the physicochemical properties and long-term stability of chitosan materials modified by mechanochemical synthesis at various levels of their structural organization including aggregates, films and three-dimensional structures. In other words, from submicron structures to macrostructures of high resolution.

**Figure 1:**
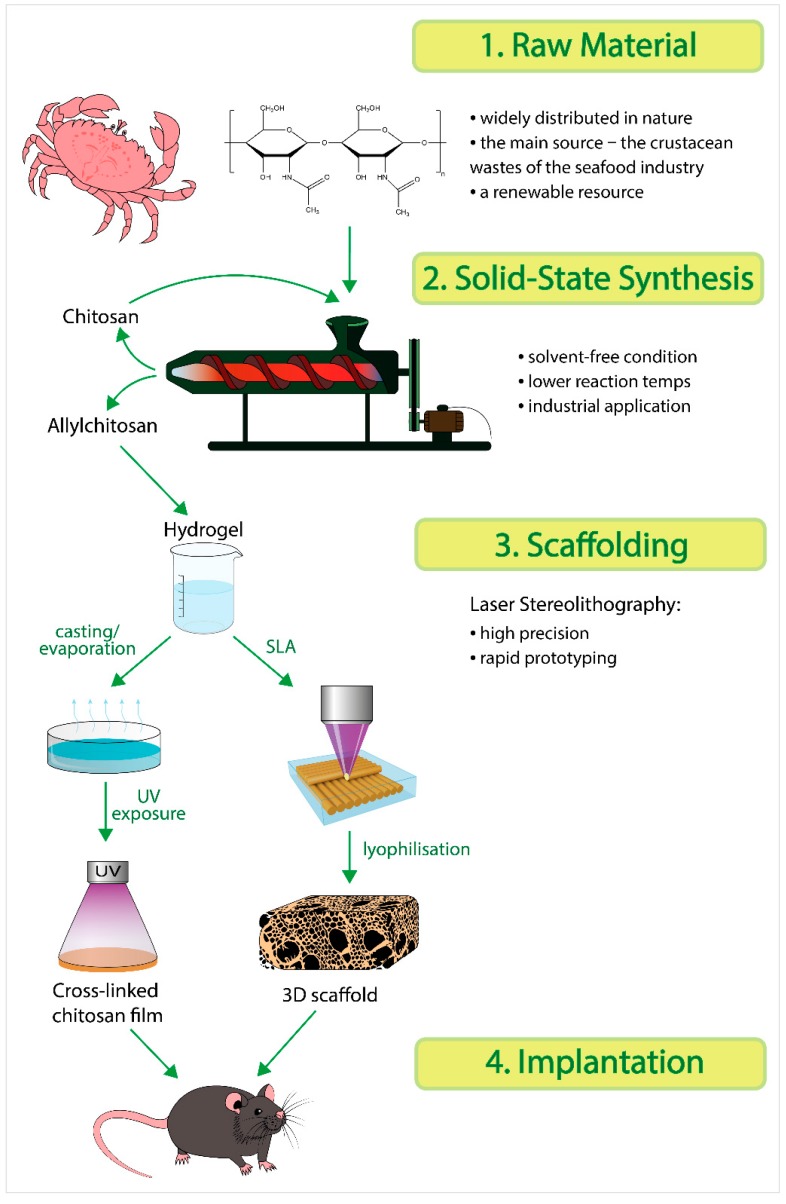
Scheme of the experimental work.

To achieve this goal, our work was divided into the following stages, according to Figure 1.

Chitosan was synthesized by deacetylation of crab chitin in a pilot-industrial extruder. Later, in the same extruder, hydrophobic fragments were introduced into the structure of chitosan. The obtained derivatives were used to prepare polymer solutions, in which forming aggregates were studied by dynamic light scattering. At the next step, photocrosslinked polymer films and three-dimensional structures (3D scaffolds) were formed. Previously, we had applied the two-photon polymerization technique to produce three-dimensional microstructures from synthesized photosensitive polysaccharides [24,25]. In this study, we used laser stereolithography, a simple and fast technique of three-dimensional prototyping requiring no expensive equipment [26,27]. The technique implies a layer-by-layer formation of a three-dimensional scaffold from a photosensitive material according to a computer-aided design (CAD) blueprint. The photosensitive material usually consists of monomers or oligomers with a photoinitiator. It is of note that, in the case of laser stereolithography, it is possible to regulate the mechanical properties of a scaffold and subsequently obtain a construct with the properties close to those of the tissues to be substituted. This is achieved by varying the parameters of laser structuring (e.g., the number of laser passes for one layer, distance both between passes and layers, laser fluence, and the scanning speed of galvanometric mirrors); regulating the number of chromophore groups in the raw material; varying the physico-mechanical characteristics of the polymer component in the composition. In addition, the long-term stability of the formed photocrosslinked films and 3D scaffolds was studied in vivo.

## 2. Results and Discussion

### 2.1. Synthesis of Allylchitosans (AC) and Their Properties

According to the NMR spectra-based calculations, the degree of substitution (per 100 polymer units) of the sample AC2 was 5% and it grew on increasing the allyl bromide (AB) in the initial mixtures: 17–20% for samples AC3, AC4 and up to 50% for the sample AC5. In the alkaline medium, O-substituted products were formed predominantly, but not selectively. The observed structure of the obtained derivatives was in total agreement with the difference in the nucleophilicity of the polymer hydroxy and amino groups under the catalytic reaction conditions. When compared to the initial polymer, the solubility of chitosan samples modified with hydrophobic allyl fragments in acetic acid (2%), a conventional solvent of chitosan, decreased insignificantly (1–2%), which allowed us to use solution methods for the preparation of such materials. Generally, our studies have shown that, for the synthesis of chitosan derivatives, mechanical activation of solid reaction mixtures is preferable since it substantially reduces the consumption of reagents, the process duration (up to several minutes), and the process temperature in contrast to a similar process in an organic solvent (isopropyl alcohol, 70 °C, 1–4 h) [28].

#### 2.1.1. Hydrodynamic Diameter of Aggregates in the Samples in Aqueous Solutions

**Figure 2:**
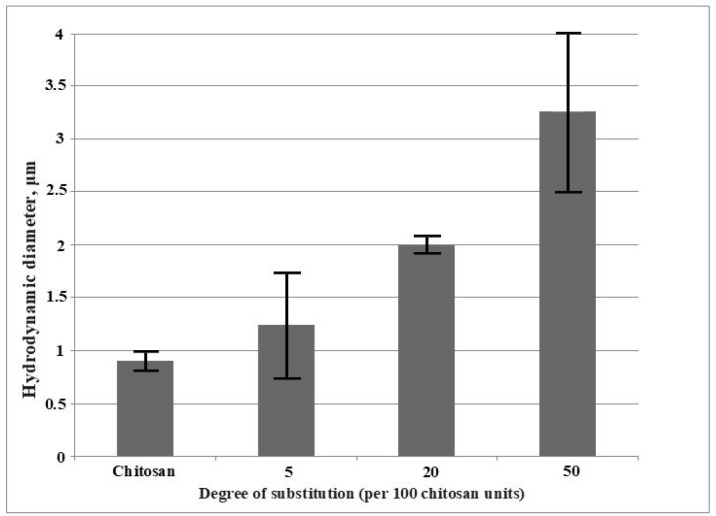
The relation between the hydrodynamic diameter and the degree of substitution of chitosan amino groups with allyl fragments. Solutions were prepared with acetic acid (2%) at a final concentration of 0.02 g/dL.

Since the use of chitosan and its derivatives is mainly associated with their aqueous solutions, it is necessary to investigate the materials’ behavior in such media. Spontaneously-formed chitosan aggregates, which can be used as drug and gene carriers [29,30], are of special interest. Figure 2 shows the relation between the hydrodynamic diameter and the degree of substitution (DS) of chitosan amino groups.

The same results for the average size of chitosan aggregates were obtained in the study by Popa-Nita et al. [31]: for 0.01 wt% chitosan solutions, aggregate sizes (diameters) ranged from 200 nm to 2000 nm. Moreover, the authors showed an increase in the aggregate sizes with an increase in the degree of acetylation, i.e., with an increase in the number of hydrophobic substituents in the chitosan structure [31]. Other studies [32,33] also showed more pronounced aggregation caused by acetamide groups left as a result of incomplete chitin deacetylation. In these articles, the aggregation was caused by either a change in the degree of protonation of dissolved macromolecules or by enhancement of hydrophobic intermolecular interactions.

However, the available data on the chitosan intermolecular aggregation are highly contradictory. The complex behavior of chitosan macromolecules in solutions is determined by the ability of their functional groups to form hydrogen bonds, both intermolecular and intramolecular, that stabilize the conformational structure. For example, some studies [34,35] showed a suppression of aggregation with an increase in the degree of acetylation, which was explained by the decrease in the chitosan crystallinity. Apparently, chitosan is prone to aggregate due to several factors, with each of them contributing to the association energy. In our experiments with dilute solutions of allylchitosan samples, dynamic light scattering revealed the formation of intermolecular lipophilic interactions. When the degree of substitution reached a significant value (sample AC5), these interactions led to the formation of aggregates, whose sizes were greater than those of the initial chitosan. Thus, the mechanical activation of solid reaction mixtures allows for the preparation of chitosan aggregates—of the required sizes by selecting the conditions for mechanochemical synthesis and varying the number of hydrophobic fragments introduced into the chitosan structure.

#### 2.1.2. Mechanical Properties of the Film Samples

Table 1 shows the effect of mechanochemical modification with allyl fragments, as well as of ultraviolet (UV) irradiation, on the mechanical properties of chitosan films. 

For the initial chitosan, the obtained values of tensile strength and elongation at break are higher than those in the study by Souza et al. [36] (σ = 13 MPa and ε = 16%) and they also exceed values for chitosan films treated with various monomers and UV irradiation by Khan et al. [37] (σ up to 29.1 MPa and ε up to 16.2%). This difference can be explained by the following factors: the origin and characteristics of chitosan source, the degree of acetylation and the molecular weight of chitosan, the solvent type, the polymer concentration in molding solutions, and the conditions for film preparation and storage [38,39].

Additional lipophilic interactions provided by allyl fragments in the structure of AC samples did not significantly contribute to their tensile strength. Moreover, UV irradiation also had no effect on the tensile strength of the films. For allylchitosan-based films, elongation at break (or plasticity) prior to UV exposure was 5–8% higher than that of the initial chitosan films. Obviously, the introduction of hydrophobic substituents into a chitosan macromolecule results in a change in the films’ crystalline morphology [40]. The UV treatment led to some decrease in the elongation at break for the AC films, especially for samples with a high degree of substitution. Such a result is expectable. UV irradiation has the ability to induce formation of cross-links in a polymer film containing unsaturated fragments. This leads to a decrease in the mobility of polymer chains, which is reflected in a lower material plasticity. The increased film hydrophobicity also causes a reduction of the water content in the film, while water acts as a plasticizer [41]. 

#### 2.1.3. X-Ray Diffraction (XRD) Analysis of Film Samples

**Figure 3:**
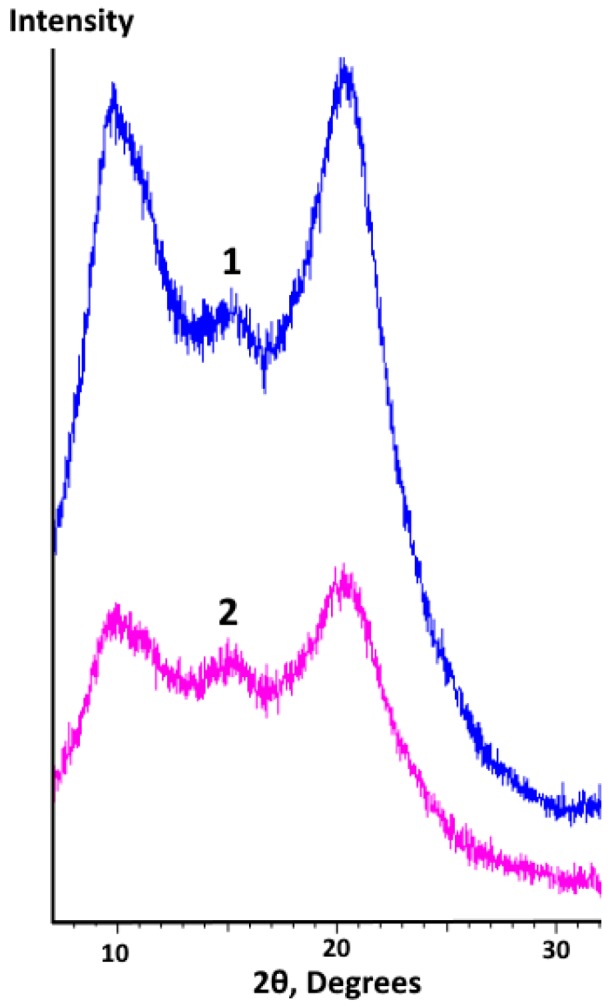
XRD analysis of allylchitosan film (sample AC2) before (spectrum 1) and after (spectrum 2) UV cross-linking.

The results of the XRD analysis for mechanochemically modified chitosan before and after UV irradiation are demonstrated in Figure 3.

When comparing the XRD data of the two samples, one can notice that cross-linking did not change the positions of the peaks. The presence of a broad intense maximum corresponding to 2θ ≈ 20 deg (d_1_ = 4.434 Å) for the mechanochemically modified chitosan is similar to other published results [36,42,43,44]. This maximum is explained by the presence of amorphous as well as “pseudocrystalline” regions (crystallites) in the polymer. These crystallites represent ordered regions where polymer chains are oriented parallel to each other. Another broad intense maximum at 2θ ≈ 10 deg (d_2_ = 8.835 Å) is assigned to the hydrated crystalline structure of chitosan, which was also observed in other studies [36,45]. The values of the interplanar distance, d_2_ and d_1_, had an approximate ratio of 2:1, which could correspond to two reflection periods between two planes of crystallites. In other words, the minimum distance between these planes was 4.434 Å. The maximum of low intensity at 2θ ≈ 15 deg (d_3_ = 5.899 Å) can also relate to the presence of crystalline regions in the structure of the initial chitosan, as was shown in several studies [43,46]. 

### 2.2. Three-Dimensional Scaffolds

**Figure 4:**
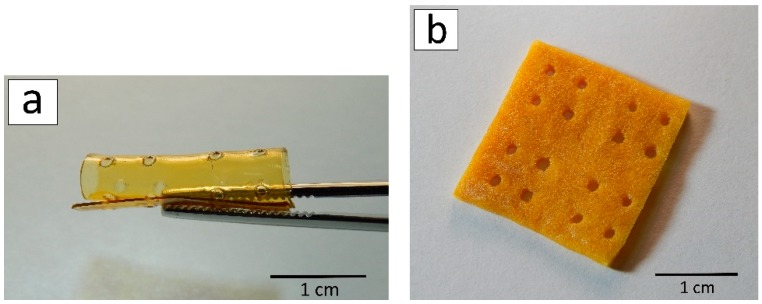
Three-dimensional scaffold after laser stereolithography (**a**) and freeze drying (**b**).

Three-dimensional scaffolds based on mechanochemically modified chitosan were successfully formed by laser stereolithography. The scaffold thicknesses of 1 mm or 2 mm were obtained, i.e., approximately six or 11 layers respectively were cross-linked layer-by-layer. The advantage of this approach to structuring chitosan derivatives is the production of three-dimensional scaffolds which have sufficient dimensions for regenerating tissue defects. After stereolithography, the scaffolds were slightly yellowish and transparent. The transparency of the allylchitosan materials allows us not only to sterilize them with UV radiation [47], but also to develop 3D structures for easy microscopy imaging [48]. In addition, the selected structuring parameters resulted in the preparation of a flexible and mechanically strong scaffold (Figure 4a). 

However, there is no internal pore architecture in such 3D scaffolds, which is a disadvantage of this structuring method since the scaffold porosity plays an important role in the diffusion of oxygen, nutrients and cellular waste [49], and supports cells’ attachment and proliferation following seeding [50]. To form allylchitosan porous structures, we used the freeze-drying method. Freeze-drying is not associated with any chemical reaction and, therefore, there are no complications with the scaffold purification from by-products. As can be seen in Figure 4b, the three-dimensional scaffold does not lose its integrity and architectonics after the process of freeze drying, and it may be rehydrated. At the same time, the scaffold loses its transparency, which is caused by phase separation during the freezing of the solvent. Freezing, in turn, forms macropores and tightly crosslinked pore walls [51,52]. The 3D scaffold becomes slightly brittle, although in general its mechanical properties are still suitable for suturing.

**Figure 5:**
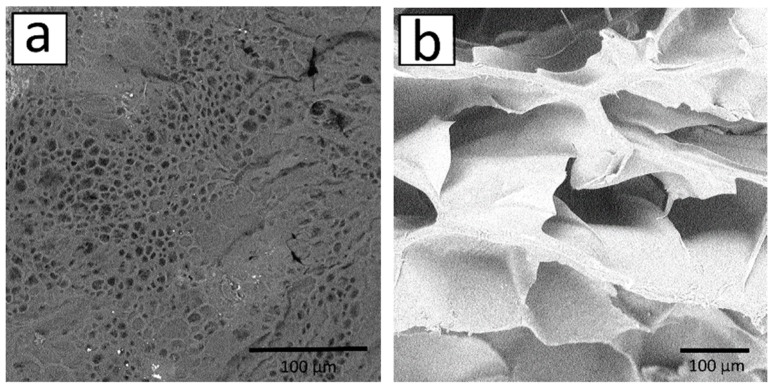
Surface SEM images of the 3D scaffold (**a**) and freeze-dried matrix (**b**), bar = 100 μm.

Evaluation of a typical pore in the scanning electron microscopy (SEM) image (Figure 5a) revealed that the pore surface occupied 30% of the total surface area. 

About 66% of pores had a size of 3–6 μm. It should be noted that the pore surface area was determined by the method of treating scaffolds after stereolithography, UV photocuring. The photocuring process was expected to additionally photo-crosslink chitosan derivatives and, as a consequence, to form more durable polymeric networks on the scaffold surface. The obtained pore sizes, as well as the presence of hydrophobic allyl fragments in the structure of chitosan, will provide a long-time efficiency of the subsequently prepared drug forms. 

Based on the evaluation of a two-dimensional section in Figure 5b, the typical pore size was 20–60 μm. Thicker parallel pore walls of the 3D scaffold are also visible in a two-dimensional section. Most likely, the walls were formed by the freeze drying of more tightly crosslinked contact regions of the previous laser-crosslinked layer with the subsequent layer.

### 2.3. Implantation of Films and Porous 3D Scaffolds based on Allylchitosans

**Figure 6:**
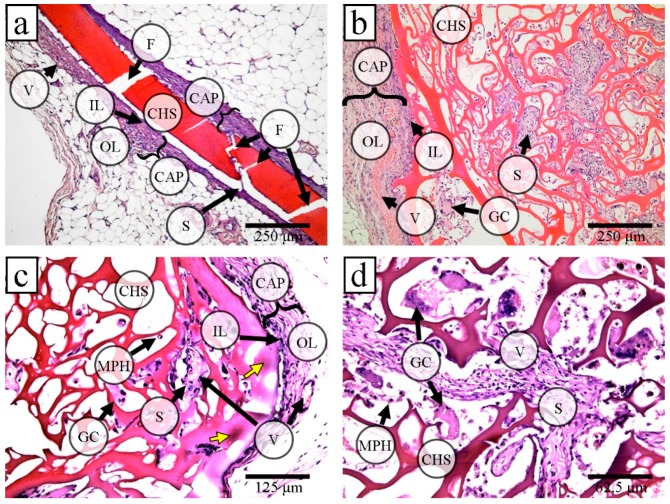
Tissue reaction to the films and the porous 3D scaffolds based on allylchitosans: the connective tissue formed the capsule (CAP) with blood vessels (V) around the implanted chitosan films (CHF) and the chitosan sponges (CHS); CAP consisted of two layers: the inner layer (IL) was an immature connective tissue (granulation tissue) with macrophages (MPH) and giant cells (GC), while the outer layer (OL) consisted of a more mature connective tissue; IL grew into the film fractures (F) and CHS pores forming connective tissue septa (S); some MPH and GC adhered to the surface of scaffolds, hematoxylin and eosin staining, simple microscopy (**a**) CHF (AC2) implantation (30 days): the CHF material was oxyphilic, 100×; (b–d)—3D scaffold implantation: (**b**) 30 days: the CHS material was oxyphilic, 100×; (**c**) a basophilic foci (yellow arrows) in the surface septa of the CHS material, 60 days, 200×; and (**d**) deep CHS sections: most of the scaffold septa were moderately basophilic, 90 days, 400×.

On day 30 after the implantation, there were no signs of biodegradation of any of the film types or 3D scaffolds. There were no significant differences in the tissue reaction to them. Tinctorial and optical properties of these scaffolds were close (Figure 6a,b; Supplementary Figures S1–S3; Figure S4a,b; Table S1). 

Connective tissue capsules surrounded all implanted scaffolds. Additionally, connective tissue carried of varying thickness and maturity. It grew into the pores of 3D scaffolds and the chitosan films fracture sites together with blood vessels. There were moderate macrophage and foreign-body giant cell reactions without any necrosis or dystrophy in surrounding tissues.

Since the film material was brittle (see Figure 6a; Supplementary Figure S1; Figure S2a,b,d,f), for the subsequent studies of the long-term stability of samples from solid-phase modified chitosan we only used porous 3D scaffolds.

On days 60 and 90 after the implantation, there were no macroscopic signs of scaffold biodegradation. On day 60 after implantation, focal changes in the tinctorial properties of the scaffolds became noticeable (Figure 6c; Supplementary Figure S5; Table S1). We clearly observed these changes in the scaffold septa (mainly near macrophages and giant cells), which had small foci of lysis of scaffold material. These changes slightly increased on day 90 (Figure 6d; Supplementary Figure S6; Tables S1 and S2). The tissue reaction to scaffolds did not significantly differ during all the post-implantation periods, except for a decrease in the maximum capsule thickness (Supplementary Figure S4c). Additionally, we observed the intensification of connective tissue ingrowth and vascularization and a giant cell reaction, the connective tissue around and inside the 3D scaffolds became more mature, grew deeper, but it did not fill the scaffold pores completely (Supplementary Tables S1 and S2). 

In summary, the implanted films and 3D scaffolds caused the foreign body reaction with the development of fibrosis without any necrosis or dystrophy in surrounding tissues, which was generally similar to the morphological results in other experiments [53,54,55,56,57,58,59].

The resorption of chitosan-based materials was also confirmed by basophilicity in other studies [54,56]. Kim et al. [56] used hematoxylin and eosin staining combined with Luxol fast blue. They suggested that, when chitosan degrades with lysozyme, the hydrolyzed β1–4 glycosidic bond results in the formation of a free anomeric hydroxyl group on the cleaved residue. Together with the C2-amino group, it is capable of forming a complex with aluminum-containing hematoxylin dye. Apparently, introduction of allyl groups does not block the access of enzymes (lysozyme, chitinase, chitosanase, glucosaminidase) capable of breaking glycosidic bonds in chitosan [60]. There is no detailed study of the relationship between the tinctorial and physicochemical characteristics of chitosan with respect to other dyes.

It should be added that in our study, there were no necrotic tissue changes or acute inflammatory reactions, reported in a number of other studies on chitosan implantation [53,55,57,58]. Most likely, we suppressed the neutrophil infiltration by introducing allyl groups in the chitosan structure, which led to an increase in the basic properties of a chitosan molecule’s fragments. This result, in turn, was similar to the proposals [57,59,61,62,63] to increase the amino group concentration (or decrease N-acetylation of chitosan) to reduce the chemotactic effect of chitosan on neutrophils.

#### Internal Structure of Film Samples

We used the wavelet analysis to characterize the microstructure of a chitosan film stained with picrosirius red. In principle, the wavelet analysis has already been suggested for applying in the clinical interpretation of histological images. For example, in the study by Haar and Daubechies [64], transform wavelets were tested as a diagnostic tool for the detection and classification of breast tumors. However, there are no studies in the field of materials science which use a wavelet transform to reveal the internal structure of scaffolds which biodegrade in vivo. 

**Figure 7:**
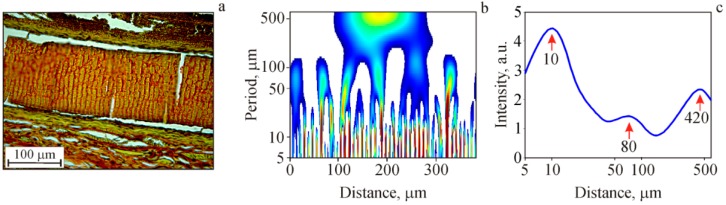
A histological section of chitosan film (**a**), a waveletgram (**b**), and the integral spatial spectrum of the structural optical inhomogeneities along the film section in the direction of its surface (**c**).

In Figure 7, we present an example of the wavelet analysis applied in our study. 

In Figure 7a, a chitosan film histological section is shown. Its structural optical inhomogeneities, yellow (picrinophilic) fragments and brown-red strips, were investigated with a constructed waveletgram. Figure 7b shows the corresponding waveletgram with vertical strips in the period range of 5 μm to 100 μm. Some of these strips join together forming a “tree structure”, while the other strips are separated from the others forming a “grass structure”. There is also a large “tree structure” in the range from 5 μm to 500 μm in the center of the waveletgram. The “tree structures” indicate that the periodicities of different scales are interrelated [65] (so-called cascade process where multiscale structures are genetically related). Using waveletgrams, an integral spatial spectrum may be plotted (Figure 7c), which characterizes the strongest periodicities for the entire processed image. For this procedure, we averaged the waveletgram along the abscissa (the distance). In the integral spatial spectrum three pronounced maxima are seen with a spatial periodicity of 10 μm, 80 μm and 420 μm. Thus, the transverse striation visualized by picrosirius red staining, depicting the areas of allylchitosan degradation, demonstrates that such areas are located periodically rather than chaotically.

When stained with picrosirius red, chitosan was colored dark-red [53,54,55], while in the study by Park et al. [66] it was picrinophilic. Most likely, the different chitosan coloration can be explained by its varying degree of degradation as well as by the diversity of chitosan crystalline forms. It is known that chitosan can exist in two distinct crystal forms, and its XR diffractogram depends on the ratio of these forms [36]. Comparing the integral spatial spectrum of the structural optical inhomogeneities along the film section (Figure 7c) and the XR diffractogram of modified chitosan films in the range of 7 < 2θ < 30 deg (see Section 2.1.3, Figure 3), one can note the "reverse" similarity of the obtained spectra. This effect indicates that crystallites are distributed throughout the entire film volume (long-range order) that apparently causes the observed structural optical inhomogeneities (the brown-red strips in picrosirius red staining). In other words, at the first stage, the degradation of film samples occurs within less ordered amorphous regions, following the increase in the chitosan degree of deacetylation due to β-1,4-glycosidic bond cleavage (depolymerization) with the subsequent N-acetyl linkage [8]. The newly released basic groups react with sulfide groups of picrosirius red. This leads to the formation of brown-red strips on day 30 after the film implantation and to the expansion of red-stained areas on day 60 after the 3D scaffold implantation.

## 3. Materials and Methods

### 3.1. Synthesis of Allylchitosans

**Figure 8:**
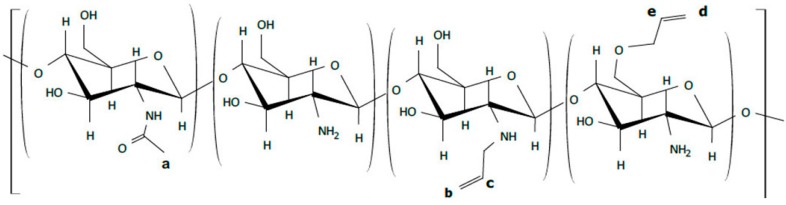
The structure of the synthesized allylchitosans.

The synthesis of unsaturated chitosan derivatives (Figure 8) was carried out by treating chitosan powders with AB in the absence of solvents under shear strain in a pilot-industrial twin-screw extruder (“Berstorff ZE 40”, Munich, Germany). 

Chitosan was prepared by mechanochemical alkaline deacetylation of crab chitin (Xiamen Fine Chemical Import and Export Co., Ltd., Xiamen, China) with a three-fold molar excess of sodium hydroxide, according to the published procedure [67], and had the following characteristics: degree of acylation (DA) = 15 mol% (evaluated by potentiometric titration of a hydrochloric solution of a sample and by NMR spectroscopy (Bruker, Billerica, MA, USA), and molecular weight (MW) = 80 kDa (evaluated by viscosimetry). The working chambers of extruder were cooled to −5 °C before adding AB to the reactive mixture. The relative number of AB molecules per elementary unit of chitosan in the mixtures was 0.5 (sample AC2), 1.0 (sample AC3), 1.5 (sample AC4), and 2.0 (sample AC5). In the case of AC3–AC5 samples, the reactive mixture contained also sodium hydroxide (2 molecules per elementary unit of chitosan), while AC2 sample was prepared using neat chitosan powder. The reaction products were purified by extraction of unreacted allyl bromide with isopropanol. The removal of alkaline impurities was performed by dialysis against distilled water until the rinsing water became neutral. The purified products were freeze-dried.

The ^1^H NMR spectra were recorded with a Bruker-Avance II-300 instrument operating at 300 MHz in D_2_O solutions at a temperature of 90 °C. A deuterated DMSO signal (δ = 2.5 ppm) was used to calibrate the chemical shift scale (Supplementary Figure S7).

The content of allyl groups in the modified chitosan was determined by measuring and comparing the integrated intensities of proton signals in the following structural fragments: >C**H**-NH_2_ (at 3.0–3.2 ppm); **H**_3_C-CONH- (Figure 8a; 1.9 ppm); **H**_2_C=CH-CH_2_-NH- (Figure 8b,c; 5.3–5.5 ppm); **H**_2_C=CH-CH_2_-O- (Figure 8d,e; 5.1–5.2 ppm). 

### 3.2. Preparation of Films and 3D Scaffolds

The films were prepared by casting solutions of polymers (2%) in acetic acid (2%) onto a plastic substrate with subsequent drying at the temperature of 20 °C for 48 h in a laminar flow cabinet. The solutions were preliminarily filtered through a membrane with a pore size of 0.45 μm, the volume was calculated based on the amount of the polymer necessary to form homogeneous films with a thickness of 100 μm. Before carrying out the mechanical tests, the films were stored in a vacuum desiccator above KOH for a week to remove the excess acid. For the biological studies, the films were immersed for 2 h in an aqueous ammonia solution (25%) for the polymer transfer from the salt to the basic form and washed with distilled water to neutral pH. 

For each type of allylchitosan, photocrosslinked films were prepared as follows: 0.5 wt% of a photoinitiator (Irgacure 2959; Ciba Specialty Chemicals, Switzerland) was added into the molding solutions of allylchitosan, and the films were illuminated for 30 min by a DRSH-500 mercury lamp. The radiation intensity at the surface of the films was 3.1 mW/cm^2^. The detailed studies of the crosslinking process of the films are given in [68].A photosensitive composition (PSC) for the preparation of 3D scaffolds by single-photon laser stereolithography was prepared as follows: an acetic acid solution (4.7 wt%) of sample AC5 was mixed with polyethylene glycol diacrylate (8 wt%; Sigma-Aldrich, MO, USA), and the mixture was stirred for 24 h at 35 °C until a homogeneous solution was achieved. Subsequently, 1 wt% of the Irgacure 2959 photoinitiator was added, and the mixture was again stirred at 35 °C for 24 h. The shelf life of PSC is three days.

The three-dimensional scaffolds were structured by laser stereolithography (LS 120, IPT RAS, Russia) according to the previous study [26]. HeCd laser (with the wavelength of 325 nm, laser power of 15 mW) was used to initiate the three-dimensional cross-linking process in the PSC. The laser beam movement along the surface of the PSC and its focusing were performed with a scanner. Then, a single scaffold layer was formed, the stage was lowered with a Z-axis motor stage to a predetermined layer thickness (200 μm), and the next scaffold layer was formed.

A 20 mm × 20 mm plate with a thickness of 1 mm or 2 mm with reach-through oval holes measuring 1 mm by 2 mm was used as a computer-aided design (CAD) blueprint. The distance between the centers of the holes was 4 mm or 6 mm (changed alternately). After the laser structuring, the samples were washed from unreacted PSC with distilled water and additionally photocured for 20 s under UV-LED light (λ = 365 nm, Epileds, Taiwan) at an intensity of 3.9 mW/cm^2^. The scaffolds were stored at +5 °C at constant humidity.

At the next stage, the obtained allylchitosan 3D scaffolds were freeze-dried using a FreeZone freeze-dryer by Labconco. When the temperature of the 3D scaffolds reached −83 °C, the pressure in the chamber was reduced to 6 μBar, and the scaffolds were left inside for one more day.

### 3.3. Characterization of Film Samples and 3D Scaffolds 

#### 3.3.1. Hydrodynamic Diameter of Samples in Aqueous Solutions

The dynamic light scattering method was applied to determine the sizes of aggregates formed by dissolving the synthesized derivatives (0.02 wt%) in acetic acid (2%). The measurements were performed with a Zetatrac (Microtrac, Inc., Montgomeryville, PA, USA) instrument using Microtrac V 10.5.3 software.

#### 3.3.2. Mechanical Properties of Film Samples

Mechanical studies were carried out using an AG-E universal testing machine (Shimadzu, Japan) at a speed of 1 mm/min. Before testing, the films were kept in a desiccator at a constant humidity of 81% above a (NH_4_)_2_SO_4_ saturated solution for a week.

#### 3.3.3. XRD Analysis

X-ray diffraction analysis was carried out with a D8 Advance diffractometer (Bruker AXS GmbH, Karlsruhe, Germany) using CuKα radiation and a "Vantec-1" detector. An allylchitosan film was fixed to a silicon single crystal substrate with liquid paraffin. The scanning step was 0.021 deg.

#### 3.3.4. SEM and the Pore Surface Area Estimation

The surface and internal structure of 3D scaffolds were characterized using a Phenom ProX scanning electron microscope (15 nm resolution; Phenom-World, Eindhoven, the Netherlands). The pore surface area was quantified with ImageJ software (National Institutes of Health, Bethesda, MD, USA [69]). 

### 3.4. Implantation

All animal experiments were performed according to the national regulations of the usage and welfare of laboratory animals and approved by the Institutional Animal Care and Use Committee in Sechenov University (Moscow, Russia) (protocol SU2018-052, approval date 12.03.2018). The laboratory rats (Wistar, average weight 450 ± 20 g) were divided into two groups. Each animal in group 1 (*n* = 5) received four film samples of different types (AC2–AC5). Animals in group 2 (*n* = 15) received 3D scaffolds (2 mm thick) and were subdivided into 3 subgroups: day 30 (*n* = 5), day 60 (*n* = 5), and day 90 (*n* = 5). The film samples and 3D scaffolds were implanted subcutaneously in the interscapular region of white rats. All manipulations were performed under anesthesia. For the premedication, a combination of 0.03 mL of Atropine solution (0.1%), 0.02 mL of diphenhydramine (10 mg/mL), and 0.05 mL of droperidol (2.5 mg/mL) was injected intramuscularly into the right thigh. General anesthesia was induced with an intramuscular injection of 0.06 mL of Zoletil (100 mg/mL) combined with 0.04 mL of Xyla (20 mg/mL) into the left thigh. The samples (0.8 cm × 0.8 cm) were implanted subcutaneously and fixed with non-absorbable sutures at the four corners of the sample to the muscles in the interscapular area. After the experiment completion, the animals were sacrificed via an intracardiac injection of 10 mL of novocaine solution (2.5%) on day 30 in group 1; on days 30, 60 and 90 in group 2. For the histological analysis, excision of a 2.0 cm × 2.0 cm interscapular area was performed.

### 3.5. Histological Analysis

Tissue fragments with implanted scaffolds (*n* = 35) were fixed in a 10% neutral phosphate-buffered formalin solution then paraffin blocks were prepared by a standard procedure. For all the samples, transverse serial sections with a thickness of 4–5 μm were stained with hematoxylin and eosin and picrosirius red for collagen fibers. We investigated the samples by light, phase-contrast, and polarization microscopies using a LEICA DM4000 B LED microscope equipped with a LEICA DFC7000 T digital video camera and LAS V4.8 software (Leica Microsystems, Wetzlar, Germany).

The connective tissue capsule thickness was measured by morphometric studies (see below). We assessed the change in tinctorial properties and material resorption, maturity of the connective tissue capsule, intensity of macrophage and giant foreign-body cell infiltration by a morphological semi-quantitative evaluation. This method was used in other similar studies [70,71]. For porous 3D scaffolds, the connective tissue ingrowth into pores and their vascularization were also assessed by a semi-quantitative evaluation.

The connective tissue capsule thickness was measured by examination of 10 selected fields of view located at an equal distance from each other at a 100× magnification. In each field the sections with the maximum, mean and minimum capsule thickness in the sample were taken into account.

In a semi-quantitative evaluation, a four-point system was used: 0 points corresponded to the absence of changes, 1 to minimal changes, 2 to moderate changes, and 3 to the maximum changes. A histological semi-quantitative scoring system for the evaluation of macrophage and foreign-body giant cell reaction to the scaffolds was based on an algorithm for semi-quantitative evaluation of inflammatory infiltration around the implantation of nanocomposites (Supplementary Table S3) [70]. Other original histological semi-quantitative scoring systems for the evaluation of chitosan scaffold’s changes and tissue reaction were original (Supplementary Tables S4–S6).

The statistical data were analyzed in GraphPad Prism 7.00 software. For each parameter, the data were tested for a normal distribution using Shapiro-Wilk test or D’Agostino and Pearson normality test. If the data fitted a normal distribution, the statistical comparison in groups was performed by the two-way ANOVA followed by Tukey’s or Sidak’s test for the comparison between groups. The correlation between different parameters was estimated with Pearson’s correlation coefficient. A difference was considered statistically significant when *p*-value < 0.05. 

#### Identification of the Internal Film Structure (Wavelet Analysis)

Sections of allylchitosan films were analyzed for periodic structures by the wavelet method [65]. In contrast to Fourier transform, wavelets allow an optimal analysis of spatial fields with a complex multiscale structure [65]. To isolate the patterns of the spatial structure at different scales, the MHAT-wavelet (Mexican HAT) was set as the basic one [72]. Moreover, such an approach could explain the mechanisms for the destruction of implanted samples. Initially, from the obtained image fragment, two one-dimensional rows were formed representing the spatial distributions of the averaged (along and across the visible surface of the chitosan film) pixel intensities. Subsequently, for each such row, a waveletgram and a spatial spectrum of structural non-uniformities were calculated [65,72].

## 4. Conclusions

Chitin and chitosan, its deacetylated product, have unique properties for use in the pharmaceutical industry and biomedicine. In the present study, we used the method of mechanochemical synthesis in a pilot setup to obtain chitosan and its derivatives with a different content of hydrophobic allyl substituents. Mechanochemical synthesis provides a high yield of products in a shorter time and at a lower temperature than a similar synthesis carried out in an organic solvent. We have shown that aggregation of chitosan derivatives becomes more pronounced with an increase in the number of hydrophobic substituents. The introduction of hydrophobic unsaturated fragments into the structure of chitosan also allows for the obtaining a photosensitive polymer, which crosslinks and forms stable three-dimensional networks under UV photocuring and laser exposure. Although photocured film samples from the obtained derivatives demonstrated no increase in the tensile strength compared to the initial chitosan, their plasticity increased. 

We have demonstrated a principal possibility of structuring the synthesized derivatives by laser stereolithography to obtain three-dimensional porous structures. Of note, the selected structuring technique allows obtaining biopolymer matrices at a centimeter scale with a high productivity, which is important for restoring large tissue defects (from 1 cm).

The long-term stability of the films and 3D scaffolds based on allylchitosan has been investigated. The histological study has shown that additional allyl fragments cause no significant changes in the tissue response for the chitosan derivatives with different degrees of substitution. No dystrophic and necrobiotic changes were detected in the surrounding tissues, which proves the biocompatibility of allylchitosan materials. We have also shown the ability of 3D scaffolds to biodegrade, with their biodegradation starting on day 60 after implantation, with its rate increased by day 90. This should be taken into consideration when producing tissue engineered scaffolds. Moreover, for the first time, we used the wavelet analysis to show that the areas of allylchitosan film degradation were periodic rather than chaotic. Comparing the results of the wavelet analysis and the XRD data, we have concluded that the degradation of the film samples occurs within less ordered amorphous regions in the polymer bulk.

## Figures and Tables

**Table 1:** Deformation-strength characteristics of allylchitosans.

Sample	DS (mol%)	Before UV Exposure			After UV Exposure		
		σ (MPa)	E (MPa)	ε (%)	σ (MPa)	E (MPa)	ε (%)
Chitosan	0	37 ± 2	1800 ± 200	18 ± 3	39 ± 3	1900 ± 200	18 ± 3
AC2	5	37 ± 2	1800 ± 200	26 ± 3	41 ± 3	1900 ± 100	21 ± 3
AC4	20	38 ± 2	2100 ± 200	25 ± 3	38 ± 2	1800 ± 200	19 ± 3
AC5	50	33 ± 2	1900 ± 200	23 ± 3	33 ± 2	1400 ± 200	17 ± 3

## Supplementary Materials

The following are available online at http://www.mdpi.com/1660-3397/17/1/48/s1, Figure S1–S2: Tissue reaction to the films based on allylchitosan (day 30); Figure S3: Tissue reaction to the 3D scaffolds based on allylchitosan (day 30); Figure S4: The data of connective tissue capsule thickness around the implanted films and 3D scaffolds based on allylchitosan; Figure S5: Tissue reaction to the 3D scaffolds based on allylchitosan (day 60); Figure S6: Tissue reaction to the 3D scaffolds based on allylchitosan (day 90); Figure S7: ^1^H NMR spectra of chitosan (1), AC2 (2) and AC5 (3); Table S1: Summary table of histological semi-quantitative analysis results; Table S2: Correlation analysis: correlations between the time after implantation and the histological findings in samples of 3D-scaffold implantations; Table S3: A histological semi-quantitative scoring system for the evaluation of macrophage and foreign-body giant cell reactions to the scaffolds; Table S4: A histological semi-quantitative scoring system for the evaluation of changes in tinctorial properties of scaffolds and scaffolds’ lysis; Table S5: A histological semi-quantitative scoring system for the evaluation of a maturity of connective tissue capsules around scaffolds; Table S6: A histological semi-quantitative scoring system for the evaluation of a connective tissue ingrowth and vascularization in pores of 3D scaffolds.
