# Supplementary material for: From Aggregates to Porous Three-Dimensional Scaffolds through a Mechanochemical Approach to Design Photosensitive Chitosan Derivatives
Source: Mar Drugs. 2019 Jan 10;17(1):48. doi: 10.3390/md17010048 (PMC6356335; doi:10.3390/md17010048)
Supplement: Supplementary file 1 [file marinedrugs-17-00048-s001.pdf]

Mar. Drugs **2019**, *17*, 48; doi:10.3390/md17010048

Supplementary Material

From Aggregates to Porous Three-Dimensional Scaffolds through a Mechanochemical Approach to Design Photosensitive Chitosan Derivatives

Kseniia N. Bardakova [1,2,*], Tatiana A. Akopova [3], Alexander V. Kurkov [1], Galina P. Goncharuk [3], Denis V. Butnaru [1], Vitaliy F. Burdukovskii [4], Artem A. Antoshin [1], Ivan A. Farion [4], Tatiana M. Zharikova [1,5], Anatoliy B. Shekhter [1], Vladimir I. Yusupov [2], Peter S. Timashev [1,2,6] and Yury A. Rochev [1,7]

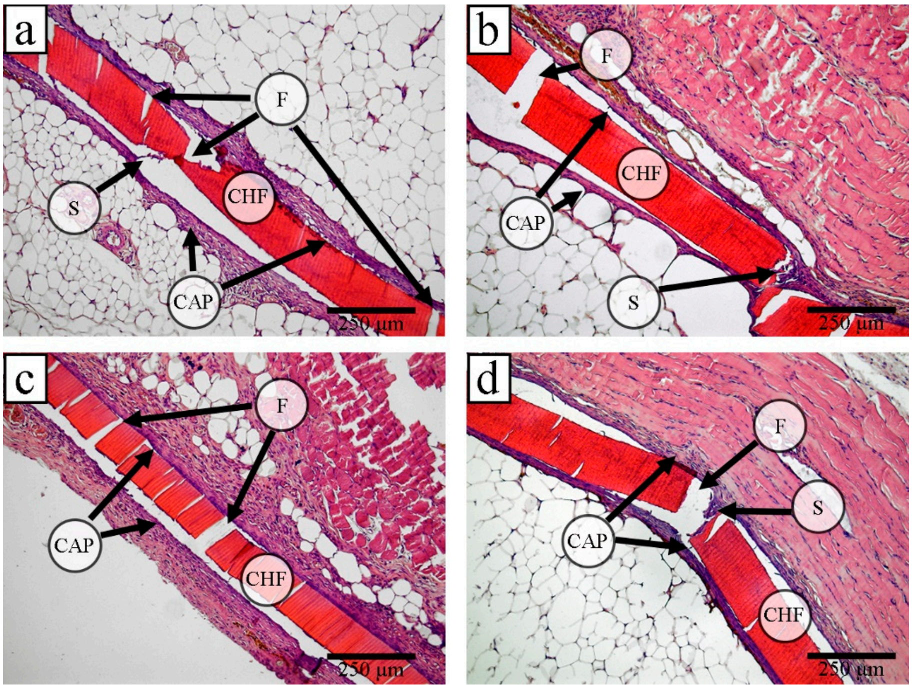

Figure S1. Tissue reaction to the films based on allylchitosan (day 30).

Note: the connective tissue formed the capsule (CAP) with blood vessels (V) around the implanted chitosan films (CHF); connective tissue grew into the film fractures (F) forming connective tissue septa (S); some macrophages (MPH) and giant cells (GC) adhered to the surface of scaffolds, the CHF material was oxyphilic, hematoxylin and eosin staining, simple microscopy, 100× (scale bar: 250 µm); (a) AC2 (b) AC3; and (c) AC4; (d) AC5.

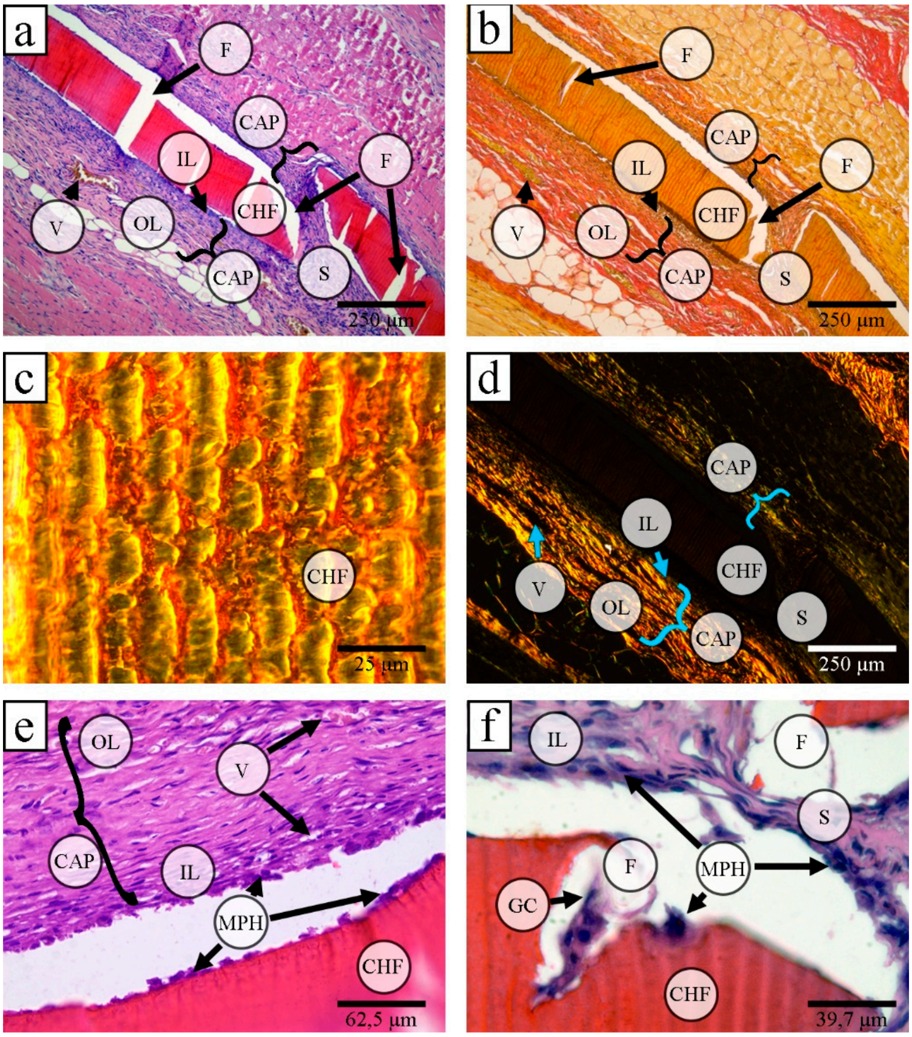

Figure S2. Tissue reaction to the films based on allylchitosan (day 30).

Note: the connective tissue formed the capsule (CAP) with blood vessels (V) around the implanted chitosan films (CHF); CAP consisted of two layers: the inner layer (IL) was an immature connective tissue (granulation tissue) with macrophages (MPH) and giant cells (GC), the outer layer (OL) consisted of a more mature connective tissue; IL grew into the film fractures (F) forming connective tissue septa (S); some MPH and GC adhered to the surface of scaffolds: (**a**) the CHF material was oxyphilic, F with displacement of the film fragments, hematoxylin and eosin staining, simple microscopy, 100× (scale bar: 250 µm); (**b**–**d**) fragments of the previous sample, picrosirius red staining: (**b**) the CHF material was picrinophilic, with the periodic structure, simple microscopy, 100× (scale bar: 250 µm); (**c**) distinct periodic structure of CHF material, phase-contrast microscopy, 1000× (scale bar: 25 µm); **d**) the CHF material was isotropic; the collagen fibers in the OL of the CAP carried of the most pronounced anisotropy (yellow and orange glow), while the collagen fibers in the IL and in the S carried of weak anisotropy (green glow), polarization microscopy, 100× (scale bar: 250 µm); (**e** and **f**) fragments of the previous sample: numerous MPH and single GC formed a lining of the IL of the capsule and adhered to the scaffold's surface, hematoxylin and eosin staining, simple microscopy: (**e**) 400× (scale bar: 62.5 µm); and (**f**) 630× (scale bar: 39.7 µm).

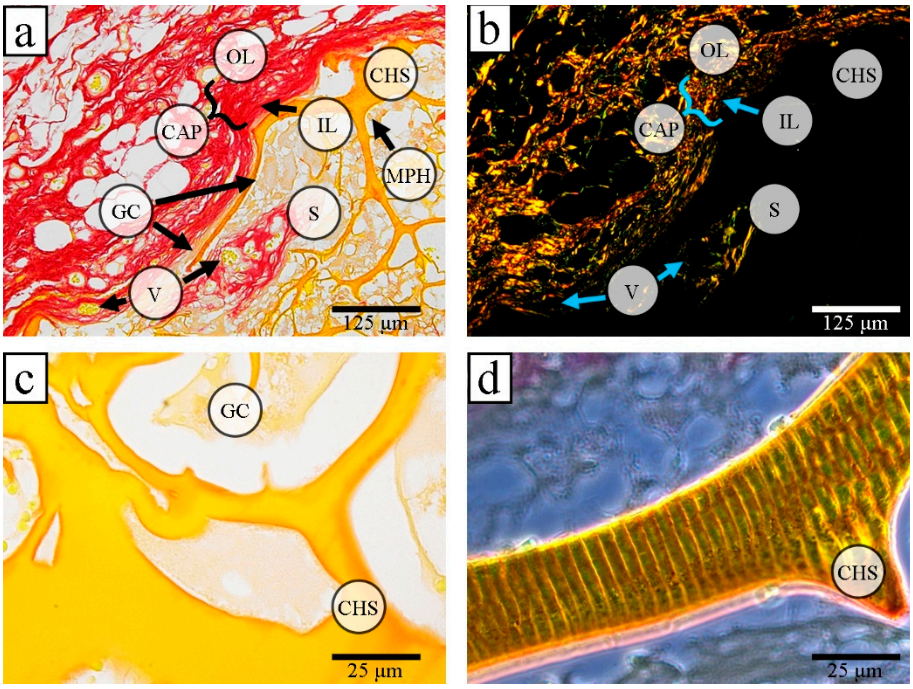

Figure S3. Tissue reaction to the 3D scaffolds based on allylchitosan (day 30).

Note: the connective tissue formed the capsule (CAP) with blood vessels (V) around the implanted chitosan sponges (CHS); CAP consisted of two layers: the inner layer (IL) was a granulation tissue with macrophages (MPH) and giant cells (GC), the outer layer (OL) consisted of a more mature connective tissue; IL grew into the pores forming connective tissue septa (S); some MPH and GC adhered to the surface of scaffolds, picrosirius red staining: (**a**) the CHS material was picrinophilic, homogeneous; the CAP and S had a significant amount of collagen fibers and a moderate vascularization, simple microscopy, 200× (scale bar: 125 μm); **b**–**d**—fragments of the previous sample: (**b**) the scaffold was isotropic; the collagen fibers in the OL of the CAP carried of the most pronounced anisotropy (yellow and orange glow), while the collagen fibers in the IL and in the S carried of weak anisotropy (green glow), polarization microscopy, 200× (scale bar: 125 μm); (**c**) GC adhered to the surface of CHS material, simple microscopy, 1000× (scale bar: 25 μm); and (**d**) there was the weak transverse striation of the scaffold's material in some septa of CHS, phase-contrast microscopy, 1000× (scale bar: 25 μm).

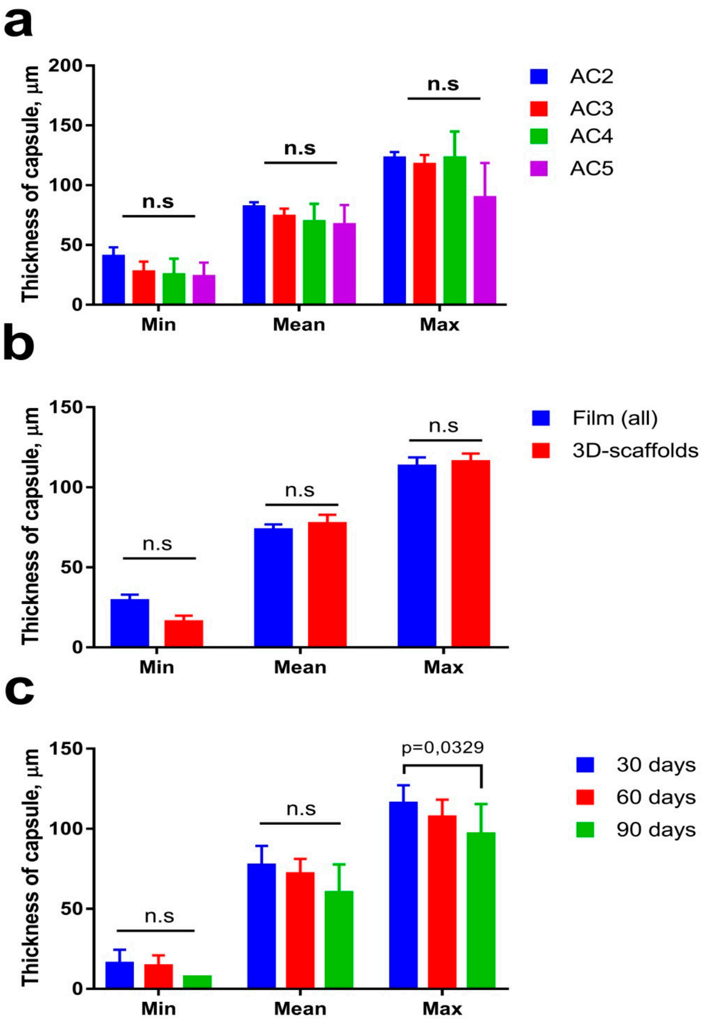

Figure S4. The data of connective tissue capsule thickness around the implanted films and 3D scaffolds based on allylchitosan.Note: (**a**) the connective tissue capsule thickness around the chitosan films (AC2–AC5), day 30, two-way ANOVA followed by Tukey's test; (**b**) the connective tissue capsule thickness around the chitosan films and 3D scaffolds, day 30, two-way ANOVA followed by Sidak's test; (**c**) the connective tissue capsule thickness around the chitosan 3D scaffolds on days 30, 60, and 90, two-way ANOVA followed by Tukey's. Data are mean ± SD or median. n.s.: no significant differences.

X-axis: the minimum (min), average (mean) and maximum (max) thickness of the capsule.

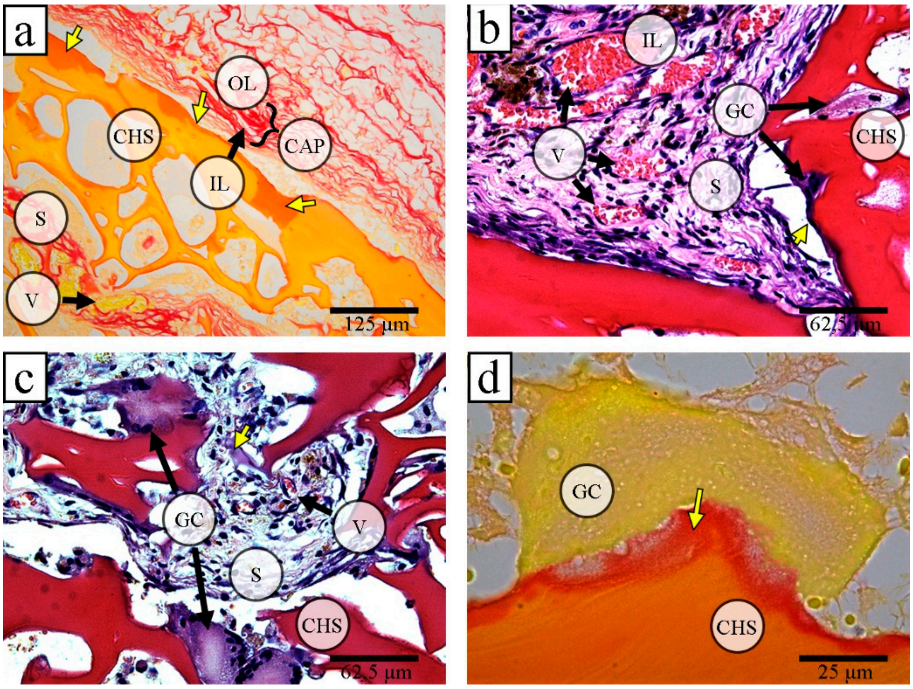

Figure S5. Tissue reaction to the 3D scaffolds based on allylchitosan (day 60).

Note: unlike day 30 there were focuses of changes in tinctorial properties of scaffolds' material (yellow arrows) in the chitosan sponge (CHS); also the inner layer (IL), the connective tissue septa (S), and the outer layer (OL) of the connective tissue capsule (CAP) consisted of a more mature connective tissue with larger blood vessels (V); giant cells (GC) were predominant, some of them adhered to the surface of scaffolds, simple microscopy: (**a**) numerous foci of red staining (yellow arrows), mainly in the surface areas of scaffold's material; numerous V in the S, picrosirius red staining, 200× (scale bar: 125 µm); (**b**) basophilic foci in the surface of scaffold's material (yellow arrow), GC on the scaffold's surface; numerous V and areas of hemosiderosis in the large S, hematoxylin and eosin staining, 400× (scale bar: 62.5 µm); (**c**) a thin septum (lysis) of scaffold with a sharp basophilia of material (yellow arrow); numerous GC adhered to the surface of the scaffold; the large S with V and a small area of hemosiderosis, hematoxylin and eosin staining, 400× (scale bar: 62.5 µm); and (**d**) the area of red staining (yellow arrow); signs of scaffold's material lysis and resorption in the place of GC adhesion, picrosirius red staining, 1000× (scale bar: 25 µm).

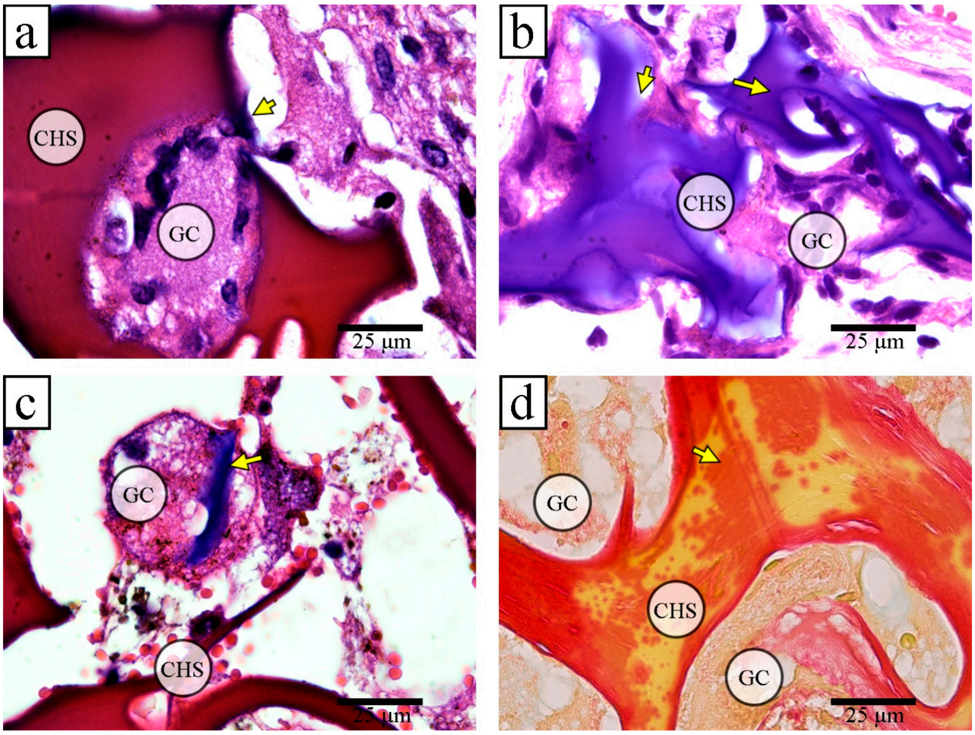

Figure S6. Tissue reaction to the 3D scaffolds based on allylchitosan (day 90).

Note: focuses of changes in tinctorial properties (yellow arrows) and lysis of the chitosan sponge (CHS) material were more prominent, than on day 60, numerous giant cells (GC), simple microscopy, 1000× (scale bars 25 µm): (**a**) basophilia in the material of the scaffold septum, which was most pronounced in the surface areas (yellow arrow); the scaffold pore was filled with a GC, hematoxylin and eosin staining; (**b**) pronounced basophilia of CHS septs (yellow arrow), some lysis areas of the scaffold's material, hematoxylin and eosin staining; (**c**) GC with a phagocytosed and partially lysed basophilic material of the CHS septum in the cytoplasm (yellow arrow), hematoxylin and eosin staining; and (**d**) areas of red staining (yellow arrows), septum lysis near the GC; some GC contained small fragments of red colored scaffold's material, picrosirius red staining.

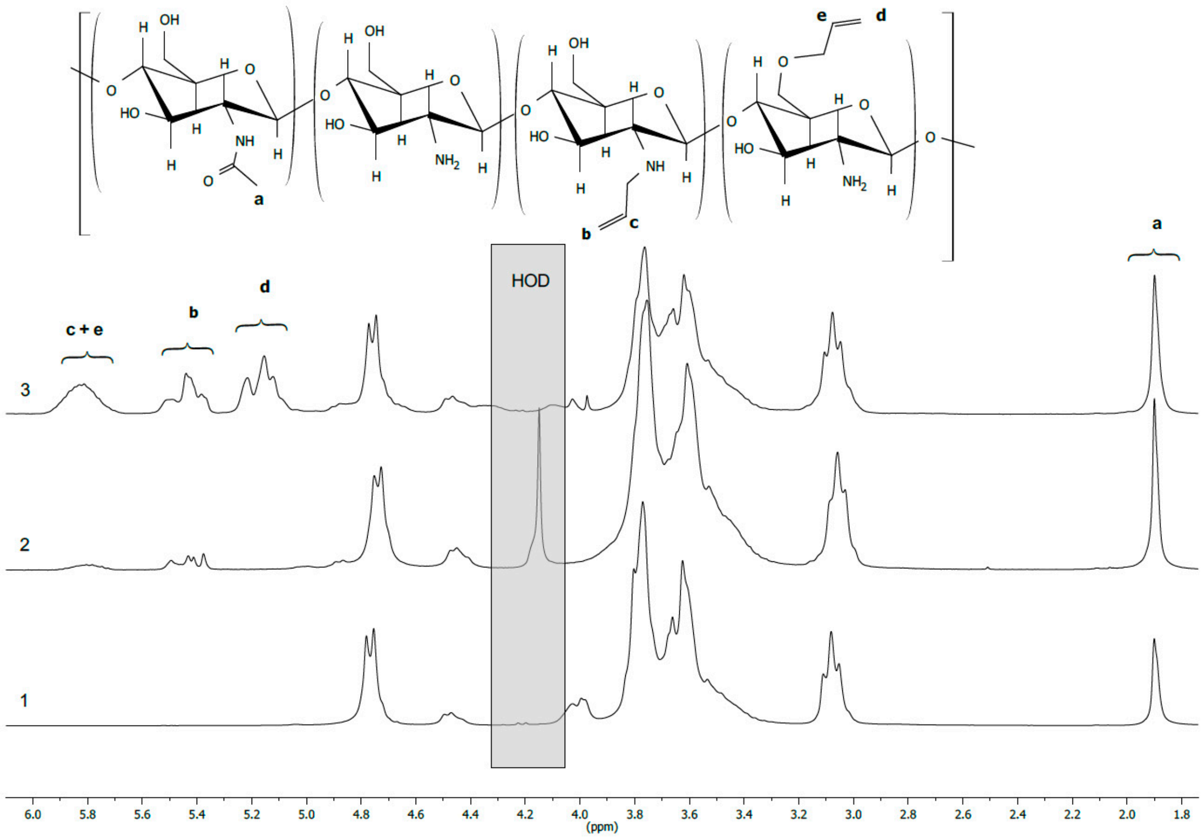

Figure S7. ^{1}H NMR spectra of chitosan (1), AC2 (2) and AC5 (3).

Table S1. Summary table of histological semiquantitative analysis results.

**Histological findings**	**Score**			
	**Films, Day 30**	**3D Scaffolds**		
		**Day 30**	**Day 60**	**Day 90**
Changes in tinctorial properties of scaffolds	0	0	1	1–2
Scaffolds' lysis	0	0	0–1	0–1
A maturity of a connective tissue capsule	2–3	2–3	3	3
Connective tissue ingrowth in pores	-	0–1	1–2	1–2
Vascularization in pores	-	0–1	1–2	1–2
The macrophage reaction	1–2	1–2	0–1	0–1
Foreign-body giant cell reaction	0–1	2	2–3	2

Table S2. Correlation analysis: correlations between the time after implantation and the histological findings in samples of 3D-scaffold implantations[1].

**Histological Findings**		**Coefficients**	***p* value**
The capsule thickness	minimum	–0.53	0.04285
	maximum	–0.54	0.03985
The degree of maturity of the connective tissue capsule		0.74	0.00166
The degree of ingrowth in scaffold pores		0.55	0.03455
The degree of vascularization in scaffold pores		0.55	0.03455
The degree of changes of the scaffold tinctorial properties		0.88	0.00002
The degree of scaffold lysis		0.52	0.04712

[1]Only significant correlations are shown

Table S3. A histological semiquantitative scoring system for the evaluation of macrophage and foreign-body giant cell reactions to the scaffolds[1].

Points	The Mean Number of Macrophages/Giant Cells on a Scaffold's Surface in 10 Random Fields of View (400×)
0	Not more than 1 cell
1	More than 1, but not more than 5 cells
2	More than 5, but not more than 11 cells
3	More than 11 cells

[1]The score system was based on an algorithm for semiquantitative evaluation of inflammatory infiltration around the implantation of nanocomposites [69].

Table S4. A histological semiquantitative scoring system for the evaluation of changes in tinctorial properties of scaffolds and scaffolds' lysis.

Points	Changes in a scaffold (changes in tinctorial properties/lysis)
0	No change or weak focal changes in less than 25% of the scaffold area
1	Weak focal changes in more than 25% of the scaffold area
2	Pronounced focal or weak diffuse changes in more than 25% of the scaffold area
3	Pronounced diffuse changes in more than 25% of the scaffold area

Table S5. A histological semiquantitative scoring system for the evaluation of a maturity of connective tissue capsules around scaffolds.

Points	Characteristics of a Capsule Around the Scaffold
0	The capsule in all areas is immature (represented exclusively by granulation tissue) or mild focal fibrosis of granulation tissue in less than 25% of the capsule area
1	Mild focal fibrosis of granulation tissue in more than 25% of the capsule area
2	Pronounced focal or weak diffuse fibrosis of granulation tissue over 25% of the capsule area
3	Pronounced diffuse fibrosis of granulation tissue in more than 25% of the capsule area

Table S6. A histological semiquantitative scoring system for the evaluation of a connective tissue ingrowth and vascularization in pores of 3D scaffolds[1].

Points	Signs of a Connective Tissue Ingrowth/Vascularization
0	All pores of the scaffold are empty or less than 25% of the surface pores contain connective tissue/blood vessels
1	More than 25% of the surface pores of a scaffold contain connective tissue/blood vessels, deep pores of a scaffold are empty
2	More than 25% of surface l pores and less than 10% of deep pores of a scaffold contain connective tissue/blood vessels
3	More than 25% of surface pores and more than 10% of deep pores of a scaffold contain connective tissue/blood vessels

[1]The pores adjacent to the outer surface were attributed to the surface pores of the 3D scaffold; the remaining pores were attributed to the deep ones.
